# Ethyl 5-(4-amino­phen­yl)isoxazole-3-carboxyl­ate

**DOI:** 10.1107/S1600536812010653

**Published:** 2012-03-17

**Authors:** Jun-Tao Zhao, Jing-Jing Qi, You-Jun Zhou, Jia-Guo Lv, Ju Zhu

**Affiliations:** a Department of Medicinal Chemistry, School of Pharmacy, Second Military Medical University, Shanghai 200433, People’s Republic of China

## Abstract

The asymmetric unit of the title compound, C_12_H_12_N_2_O_3_, contains two mol­ecules in which the benzene and isoxazole rings are almost coplanar, the dihedral angles between their mean planes being 1.76 (9) and 5.85 (8)°. The two mol­ecules inter­act with each other *via* N—H⋯N and N—H⋯O hydrogen bonds, which link the mol­ecules into layers parallel to the *ac* plane. The layers stack in a parallel mode with an inter­layer distance of 3.36 (7) Å.

## Related literature

For the synthesis and biological activity of soxazoles, see; Silva *et al.* (2002 ▶); Changtam *et al.* (2010 ▶); Patel *et al.* (2010 ▶); Barceló *et al.* (2007 ▶); Yamamoto *et al.* (2007 ▶); Mao *et al.* (2010 ▶). For their structure–activity relationships, see: Andrzejak *et al.* (2011 ▶); Becht *et al.* (2006 ▶); Veronese *et al.* (1997 ▶). For our research in this area, see: Qi *et al.* (2011 ▶).
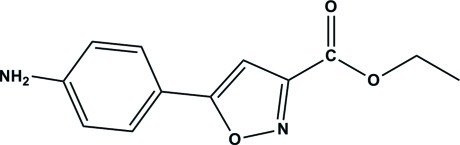


## Experimental

### 

#### Crystal data

C_12_H_12_N_2_O_3_
*M* *_r_* = 232.24
Triclinic, 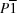

*a* = 7.591 (2) Å
*b* = 11.303 (4) Å
*c* = 13.818 (4) Å
α = 88.155 (4)°
β = 87.008 (4)°
γ = 86.233 (4)°
*V* = 1181.0 (6) Å^3^
*Z* = 4
Mo *K*α radiation
μ = 0.10 mm^−1^
*T* = 293 K
0.15 × 0.09 × 0.08 mm

#### Data collection

Bruker SMART CCD area-detector diffractometer
Absorption correction: multi-scan (*SADABS*; Bruker, 1999 ▶) *T*_min_ = 0.986, *T*_max_ = 0.992
4901 measured reflections
4074 independent reflections
2636 reflections with *I* > 2σ(*I*)
*R*_int_ = 0.022

#### Refinement

*R*[*F* ^2^ > 2σ(*F* ^2^)] = 0.044
*wR*(*F* ^2^) = 0.122
*S* = 0.98
4074 reflections
308 parameters
H-atom parameters constrained
Δρ_max_ = 0.18 e Å^−3^
Δρ_min_ = −0.20 e Å^−3^

### 

Data collection: *SMART* (Bruker, 1999 ▶); cell refinement: *SAINT* (Bruker, 1999 ▶); data reduction: *SAINT*; program(s) used to solve structure: *SHELXS97* (Sheldrick, 2008 ▶); program(s) used to refine structure: *SHELXL97* (Sheldrick, 2008 ▶); molecular graphics: *SHELXTL* (Sheldrick, 2008 ▶); software used to prepare material for publication: *SHELXL97*.

## Supplementary Material

Crystal structure: contains datablock(s) I, global. DOI: 10.1107/S1600536812010653/bv2198sup1.cif

Structure factors: contains datablock(s) I. DOI: 10.1107/S1600536812010653/bv2198Isup2.hkl

Supplementary material file. DOI: 10.1107/S1600536812010653/bv2198Isup3.cdx

Supplementary material file. DOI: 10.1107/S1600536812010653/bv2198Isup4.cml

Additional supplementary materials:  crystallographic information; 3D view; checkCIF report

## Figures and Tables

**Table 1:** Hydrogen-bond geometry (Å, °)

*D*—H⋯*A*	*D*—H	H⋯*A*	*D*⋯*A*	*D*—H⋯*A*
N3—H3*C*⋯N2^i^	0.86	2.44	3.243 (3)	157
N3—H3*B*⋯O6^ii^	0.86	2.62	3.396 (2)	150
N1—H1*B*⋯N4	0.86	2.44	3.255 (3)	159
N1—H1*A*⋯O3^iii^	0.86	2.63	3.394 (3)	149

Symmetry codes: (i) 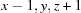; (ii) 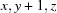; (iii) 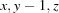.

**Fig. 1.**
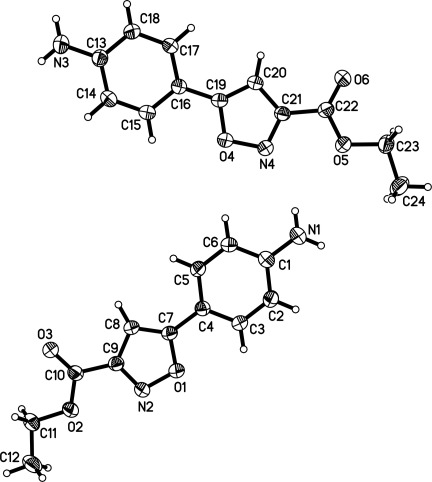
The molecular structure of the title compound, with atom labels and 30% probability displacement ellipsoids for non-H atoms.

**Fig. 2.**
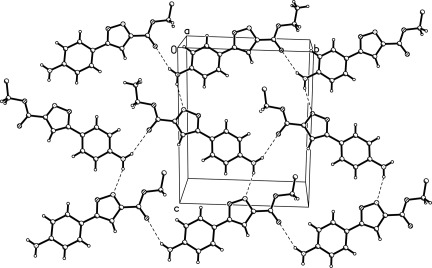
Intermolecular N—H···N and N—H···O contacts forming a supramolecular sheet.

## supplementary crystallographic information

### Comment

Isoxazoles are important compounds possessing pharmaceutical properties. Extensive investigation on the crystal structures of isoxazoles helps disclose their structure-activity relationship (Veronese *et al.* (1997); Becht *et al.* (2006); Andrzejak *et al.* (2011)). In a continuation of our research (Qi *et al.* (2011)), herein, we report the crystal structure of the title isoxazole derivative. The asymmetric unit of the title compound, C_12_H_12_N_2_O_3_, contains two planar molecules. In the molecular structure, (I) (Fig. 1), the dihedral angle between the isoxazole ring C7/C8/C9/N2/O1 and phenyl ring C1/C2/C3/C4/C5/C6 is 1.76 (9)° for molecule 1. The amino-group of the benzene ring is nearly into the same plane (r.m.s. deviation = 0.034 Å) as is usual for amino groups attached to aromatic rings. The COOEt group of the isoxazole ring is also in the same plane. The dihedral angle between the carboxylate and the isoxazole ring is 0.92 (13)°. For molecule 2, the dihedral angle for the isoxazole ring C19/C20/C21/N4/O4 and phenyl ring C13/C14/C15/C16/C17/C18 is 5.85 (8)°, which is slightly larger than molecule 1. The carboxylate group also has a little distortion with the larger dihedral angle between the carboxylate and the isoxazole ring being 1.58 (11)°. The two molecules interact with each other by strong N—H···N and N—H···O hydrogen bonds, which link the molecules into a layer (Fig. 2, Table 1). The layers then stack in parallel mode with the interlayer distance of 3.36 (7) Å.

### Experimental

After a reaction of 4-nitroacetophenone and diethyl oxalate in a basic solution of ethanol for 2hrs, then add acetic acid to neutralize the solution from former reaction to obtain yellow solids. The solids were collected and reacted with hydroxylamine hydrochloride in ethanol at reflux for 4 hrs to form yellow products which were then reduced with stannous chloride in ethyl acetate to yield the title compound.

### Refinement

H atoms were placed in geometrically idealized positions, and refined as riding on their parent atoms, with C—H distances fixed to 0.93 Å (aromatic CH), 0.97 (CH_2_) with *U*_iso_ = 1.2Ueq(C) and 0.96 Å (methyl CH_3_) with *U*_iso_ = 1.5Ueq(C). The N—H distances are fixed to 0.86 Å (*U*_iso_ = 1.5Ueq(N)).

### Crystal data

**Table:**

C_12_H_12_N_2_O_3_	*Z* = 4
*M**_r_* = 232.24	*F*(000) = 488
Triclinic, *P*1	*D*_x_ = 1.306 Mg m^−^^3^
Hall symbol: -P 1	Mo *K*α radiation, λ = 0.71073 Å
*a* = 7.591 (2) Å	Cell parameters from 1005 reflections
*b* = 11.303 (4) Å	θ = 3.0–24.5°
*c* = 13.818 (4) Å	µ = 0.10 mm^−^^1^
α = 88.155 (4)°	*T* = 293 K
β = 87.008 (4)°	Block, yellow
γ = 86.233 (4)°	0.15 × 0.09 × 0.08 mm
*V* = 1181.0 (6) Å^3^	

### Data collection

**Table:**

Bruker SMART CCD area-detector diffractometer	4074 independent reflections
Radiation source: fine-focus sealed tube	2636 reflections with *I* > 2σ(*I*)
Graphite monochromator	*R*_int_ = 0.022
phi and ω scans	θ_max_ = 25.0°, θ_min_ = 1.5°
Absorption correction: multi-scan (*SADABS*; Bruker, 1999)	*h* = −9→8
*T*_min_ = 0.986, *T*_max_ = 0.992	*k* = −12→13
4901 measured reflections	*l* = −14→16

### Refinement

**Table:**

Refinement on *F*^2^	Secondary atom site location: difference Fourier map
Least-squares matrix: full	Hydrogen site location: inferred from neighbouring sites
*R*[*F*^2^ > 2σ(*F*^2^)] = 0.044	H-atom parameters constrained
*wR*(*F*^2^) = 0.122	*w* = 1/[σ^2^(*F*_o_^2^) + (0.0658*P*)^2^] where *P* = (*F*_o_^2^ + 2*F*_c_^2^)/3
*S* = 0.98	(Δ/σ)_max_ = 0.001
4074 reflections	Δρ_max_ = 0.18 e Å^−^^3^
308 parameters	Δρ_min_ = −0.20 e Å^−^^3^
0 restraints	Extinction correction: *SHELXL97* (Sheldrick, 2008), Fc^*^=kFc[1+0.001xFc^2^λ^3^/sin(2θ)]^-1/4^
Primary atom site location: structure-invariant direct methods	Extinction coefficient: 0.0095 (16)

### Special details

**Table:**

Geometry. All e.s.d.'s (except the e.s.d. in the dihedral angle between two l.s. planes) are estimated using the full covariance matrix. The cell e.s.d.'s are taken into account individually in the estimation of e.s.d.'s in distances, angles and torsion angles; correlations between e.s.d.'s in cell parameters are only used when they are defined by crystal symmetry. An approximate (isotropic) treatment of cell e.s.d.'s is used for estimating e.s.d.'s involving l.s. planes.
Refinement. Refinement of *F*^2^ against ALL reflections. The weighted *R*-factor *wR* and goodness of fit *S* are based on *F*^2^, conventional *R*-factors *R* are based on *F*, with *F* set to zero for negative *F*^2^. The threshold expression of *F*^2^ > σ(*F*^2^) is used only for calculating *R*-factors(gt) *etc*. and is not relevant to the choice of reflections for refinement. *R*-factors based on *F*^2^ are statistically about twice as large as those based on *F*, and *R*- factors based on ALL data will be even larger.

### Fractional atomic coordinates and isotropic or equivalent isotropic displacement parameters (Å^2^)

**Table:**

	*x*	*y*	*z*	*U*_iso_*/*U*_eq_	
N1	0.5335 (2)	−0.04171 (15)	0.21800 (13)	0.0772 (6)	
H1A	0.5476	−0.1051	0.1850	0.093*	
H1B	0.4906	−0.0450	0.2768	0.093*	
N2	0.8174 (2)	0.50932 (15)	−0.07787 (12)	0.0667 (5)	
O1	0.7833 (2)	0.39375 (12)	−0.04673 (10)	0.0676 (4)	
O2	0.85208 (17)	0.74084 (11)	−0.09551 (9)	0.0599 (4)	
O3	0.7322 (2)	0.76842 (12)	0.05446 (10)	0.0754 (5)	
C1	0.5794 (3)	0.06453 (17)	0.17699 (14)	0.0546 (5)	
C2	0.6487 (3)	0.07114 (18)	0.08224 (14)	0.0588 (5)	
H2B	0.6668	0.0021	0.0473	0.071*	
C3	0.6913 (3)	0.17713 (17)	0.03879 (14)	0.0568 (5)	
H3A	0.7380	0.1788	−0.0248	0.068*	
C4	0.6653 (2)	0.28223 (16)	0.08896 (13)	0.0487 (5)	
C5	0.5955 (3)	0.27542 (17)	0.18384 (14)	0.0559 (5)	
H5A	0.5774	0.3444	0.2189	0.067*	
C6	0.5525 (3)	0.16923 (18)	0.22717 (14)	0.0585 (5)	
H6A	0.5050	0.1675	0.2906	0.070*	
C7	0.7110 (2)	0.39554 (17)	0.04488 (13)	0.0483 (5)	
C8	0.6983 (2)	0.50841 (17)	0.07360 (13)	0.0517 (5)	
H8A	0.6549	0.5362	0.1334	0.062*	
C9	0.7640 (2)	0.57479 (17)	−0.00509 (13)	0.0492 (5)	
C10	0.7795 (3)	0.70477 (18)	−0.01060 (14)	0.0530 (5)	
C11	0.8622 (3)	0.86764 (17)	−0.11021 (14)	0.0612 (6)	
H11A	0.9401	0.8978	−0.0647	0.073*	
H11B	0.7460	0.9077	−0.1005	0.073*	
C12	0.9325 (3)	0.8886 (2)	−0.21169 (16)	0.0807 (7)	
H12A	0.9406	0.9722	−0.2240	0.121*	
H12B	0.8544	0.8583	−0.2560	0.121*	
H12C	1.0477	0.8489	−0.2203	0.121*	
N3	0.0413 (3)	0.56811 (15)	0.72204 (13)	0.0812 (6)	
H3B	0.0532	0.6332	0.6891	0.097*	
H3C	−0.0011	0.5699	0.7810	0.097*	
N4	0.3229 (2)	0.01690 (15)	0.42390 (11)	0.0633 (5)	
O4	0.2962 (2)	0.13256 (12)	0.45691 (9)	0.0659 (4)	
O5	0.33623 (18)	−0.21554 (12)	0.40201 (9)	0.0609 (4)	
O6	0.2162 (2)	−0.24455 (13)	0.55129 (10)	0.0821 (5)	
C13	0.0896 (3)	0.46186 (17)	0.68074 (14)	0.0552 (5)	
C14	0.1581 (3)	0.45657 (18)	0.58602 (14)	0.0600 (6)	
H14A	0.1750	0.5268	0.5508	0.072*	
C15	0.2013 (3)	0.35068 (17)	0.54298 (14)	0.0561 (5)	
H15A	0.2459	0.3504	0.4790	0.067*	
C16	0.1801 (2)	0.24361 (16)	0.59284 (13)	0.0480 (5)	
C17	0.1131 (3)	0.24851 (17)	0.68872 (13)	0.0539 (5)	
H17A	0.0984	0.1781	0.7240	0.065*	
C18	0.0685 (3)	0.35428 (17)	0.73236 (13)	0.0554 (5)	
H18A	0.0241	0.3548	0.7964	0.066*	
C19	0.2207 (2)	0.13038 (16)	0.54784 (13)	0.0476 (5)	
C20	0.1997 (3)	0.01638 (16)	0.57400 (13)	0.0538 (5)	
H20A	0.1517	−0.0124	0.6327	0.065*	
C21	0.2648 (2)	−0.04958 (16)	0.49515 (13)	0.0478 (5)	
C22	0.2698 (3)	−0.18029 (18)	0.48701 (14)	0.0541 (5)	
C23	0.3394 (3)	−0.34175 (17)	0.38527 (15)	0.0625 (6)	
H23A	0.2214	−0.3695	0.3949	0.075*	
H23B	0.4157	−0.3853	0.4301	0.075*	
C24	0.4079 (3)	−0.3605 (2)	0.28296 (16)	0.0858 (8)	
H24A	0.4114	−0.4435	0.2695	0.129*	
H24B	0.5248	−0.3330	0.2744	0.129*	
H24C	0.3314	−0.3171	0.2393	0.129*	

### Atomic displacement parameters (Å^2^)

**Table:**

	*U*^11^	*U*^22^	*U*^33^	*U*^12^	*U*^13^	*U*^23^
N1	0.1088 (16)	0.0514 (11)	0.0701 (12)	−0.0117 (10)	0.0120 (11)	0.0054 (9)
N2	0.0963 (14)	0.0488 (11)	0.0533 (10)	−0.0078 (9)	0.0149 (9)	0.0033 (8)
O1	0.0967 (12)	0.0534 (9)	0.0509 (8)	−0.0076 (7)	0.0199 (7)	−0.0033 (6)
O2	0.0678 (10)	0.0536 (9)	0.0568 (8)	−0.0068 (7)	0.0106 (7)	0.0064 (6)
O3	0.1151 (13)	0.0534 (9)	0.0553 (9)	−0.0011 (8)	0.0141 (9)	−0.0025 (7)
C1	0.0528 (13)	0.0533 (13)	0.0576 (12)	−0.0051 (9)	−0.0034 (10)	0.0033 (10)
C2	0.0649 (14)	0.0523 (13)	0.0592 (13)	−0.0035 (10)	0.0018 (10)	−0.0088 (10)
C3	0.0693 (15)	0.0539 (13)	0.0465 (11)	−0.0036 (10)	0.0044 (10)	−0.0044 (9)
C4	0.0496 (12)	0.0488 (12)	0.0472 (11)	−0.0020 (9)	−0.0003 (9)	−0.0007 (9)
C5	0.0620 (14)	0.0509 (12)	0.0542 (12)	−0.0048 (10)	0.0088 (10)	−0.0077 (9)
C6	0.0676 (14)	0.0577 (13)	0.0492 (11)	−0.0079 (10)	0.0102 (10)	−0.0008 (10)
C7	0.0473 (12)	0.0544 (13)	0.0423 (10)	−0.0010 (9)	0.0023 (8)	−0.0016 (9)
C8	0.0592 (13)	0.0518 (13)	0.0433 (11)	−0.0030 (9)	0.0069 (9)	−0.0031 (9)
C9	0.0488 (12)	0.0525 (12)	0.0456 (11)	−0.0009 (9)	0.0011 (9)	−0.0010 (9)
C10	0.0570 (13)	0.0518 (12)	0.0492 (12)	−0.0001 (9)	−0.0022 (10)	0.0050 (10)
C11	0.0560 (13)	0.0543 (13)	0.0723 (14)	−0.0050 (10)	0.0010 (11)	0.0117 (10)
C12	0.0848 (18)	0.0749 (16)	0.0781 (16)	−0.0020 (13)	0.0148 (13)	0.0259 (12)
N3	0.1247 (18)	0.0499 (12)	0.0672 (12)	−0.0007 (11)	0.0104 (11)	−0.0075 (9)
N4	0.0878 (13)	0.0491 (11)	0.0515 (10)	−0.0044 (9)	0.0132 (9)	−0.0037 (8)
O4	0.0948 (11)	0.0497 (9)	0.0510 (8)	−0.0054 (7)	0.0186 (7)	0.0013 (6)
O5	0.0722 (10)	0.0545 (9)	0.0560 (9)	−0.0103 (7)	0.0109 (7)	−0.0109 (6)
O6	0.1332 (15)	0.0530 (9)	0.0592 (9)	−0.0192 (9)	0.0197 (9)	−0.0005 (7)
C13	0.0627 (14)	0.0497 (12)	0.0533 (12)	−0.0020 (10)	−0.0045 (10)	−0.0040 (9)
C14	0.0734 (15)	0.0504 (13)	0.0558 (12)	−0.0088 (10)	0.0014 (11)	0.0075 (10)
C15	0.0680 (14)	0.0541 (13)	0.0452 (11)	−0.0061 (10)	0.0073 (10)	0.0032 (9)
C16	0.0503 (12)	0.0468 (12)	0.0462 (11)	−0.0025 (9)	0.0012 (9)	0.0002 (9)
C17	0.0649 (14)	0.0476 (12)	0.0482 (11)	−0.0036 (9)	0.0033 (10)	0.0058 (9)
C18	0.0657 (14)	0.0544 (13)	0.0450 (11)	−0.0012 (10)	0.0033 (9)	−0.0019 (9)
C19	0.0469 (12)	0.0542 (12)	0.0409 (10)	−0.0039 (9)	0.0028 (8)	0.0024 (9)
C20	0.0666 (14)	0.0495 (12)	0.0446 (11)	−0.0096 (10)	0.0093 (10)	0.0018 (9)
C21	0.0493 (12)	0.0505 (12)	0.0434 (11)	−0.0078 (9)	0.0028 (9)	0.0008 (9)
C22	0.0595 (13)	0.0561 (13)	0.0470 (12)	−0.0079 (10)	0.0026 (10)	−0.0062 (10)
C23	0.0619 (14)	0.0555 (14)	0.0712 (14)	−0.0097 (10)	0.0025 (11)	−0.0152 (10)
C24	0.0895 (19)	0.0888 (18)	0.0801 (17)	−0.0188 (14)	0.0221 (14)	−0.0357 (14)

### Geometric parameters (Å, º)

**Table:**

N1—C1	1.369 (2)	N3—C13	1.366 (2)
N1—H1A	0.8600	N3—H3B	0.8600
N1—H1B	0.8600	N3—H3C	0.8600
N2—C9	1.300 (2)	N4—C21	1.296 (2)
N2—O1	1.398 (2)	N4—O4	1.397 (2)
O1—C7	1.354 (2)	O4—C19	1.354 (2)
O2—C10	1.333 (2)	O5—C22	1.317 (2)
O2—C11	1.448 (2)	O5—C23	1.451 (2)
O3—C10	1.195 (2)	O6—C22	1.201 (2)
C1—C2	1.387 (3)	C13—C14	1.384 (3)
C1—C6	1.389 (3)	C13—C18	1.404 (3)
C2—C3	1.373 (3)	C14—C15	1.367 (3)
C2—H2B	0.9300	C14—H14A	0.9300
C3—C4	1.392 (3)	C15—C16	1.388 (3)
C3—H3A	0.9300	C15—H15A	0.9300
C4—C5	1.390 (2)	C16—C17	1.396 (2)
C4—C7	1.455 (2)	C16—C19	1.449 (3)
C5—C6	1.375 (3)	C17—C18	1.371 (3)
C5—H5A	0.9300	C17—H17A	0.9300
C6—H6A	0.9300	C18—H18A	0.9300
C7—C8	1.344 (3)	C19—C20	1.344 (2)
C8—C9	1.391 (2)	C20—C21	1.391 (2)
C8—H8A	0.9300	C20—H20A	0.9300
C9—C10	1.481 (3)	C21—C22	1.483 (3)
C11—C12	1.491 (3)	C23—C24	1.497 (3)
C11—H11A	0.9700	C23—H23A	0.9700
C11—H11B	0.9700	C23—H23B	0.9700
C12—H12A	0.9600	C24—H24A	0.9600
C12—H12B	0.9600	C24—H24B	0.9600
C12—H12C	0.9600	C24—H24C	0.9600
			
C1—N1—H1A	120.0	C13—N3—H3B	120.0
C1—N1—H1B	120.0	C13—N3—H3C	120.0
H1A—N1—H1B	120.0	H3B—N3—H3C	120.0
C9—N2—O1	104.72 (15)	C21—N4—O4	104.79 (15)
C7—O1—N2	109.27 (14)	C19—O4—N4	109.59 (13)
C10—O2—C11	116.18 (15)	C22—O5—C23	116.53 (15)
N1—C1—C2	120.60 (18)	N3—C13—C14	121.13 (18)
N1—C1—C6	121.43 (19)	N3—C13—C18	121.11 (19)
C2—C1—C6	117.93 (18)	C14—C13—C18	117.75 (18)
C3—C2—C1	121.62 (18)	C15—C14—C13	121.60 (18)
C3—C2—H2B	119.2	C15—C14—H14A	119.2
C1—C2—H2B	119.2	C13—C14—H14A	119.2
C2—C3—C4	120.66 (18)	C14—C15—C16	121.32 (18)
C2—C3—H3A	119.7	C14—C15—H15A	119.3
C4—C3—H3A	119.7	C16—C15—H15A	119.3
C5—C4—C3	117.62 (17)	C15—C16—C17	117.28 (17)
C5—C4—C7	120.65 (16)	C15—C16—C19	122.22 (17)
C3—C4—C7	121.72 (17)	C17—C16—C19	120.48 (16)
C6—C5—C4	121.65 (18)	C18—C17—C16	121.82 (17)
C6—C5—H5A	119.2	C18—C17—H17A	119.1
C4—C5—H5A	119.2	C16—C17—H17A	119.1
C5—C6—C1	120.52 (19)	C17—C18—C13	120.22 (18)
C5—C6—H6A	119.7	C17—C18—H18A	119.9
C1—C6—H6A	119.7	C13—C18—H18A	119.9
C8—C7—O1	108.23 (16)	C20—C19—O4	107.67 (16)
C8—C7—C4	134.92 (17)	C20—C19—C16	135.29 (17)
O1—C7—C4	116.84 (16)	O4—C19—C16	117.04 (15)
C7—C8—C9	105.37 (17)	C19—C20—C21	105.79 (16)
C7—C8—H8A	127.3	C19—C20—H20A	127.1
C9—C8—H8A	127.3	C21—C20—H20A	127.1
N2—C9—C8	112.39 (18)	N4—C21—C20	112.16 (17)
N2—C9—C10	120.72 (17)	N4—C21—C22	120.80 (17)
C8—C9—C10	126.88 (18)	C20—C21—C22	127.03 (17)
O3—C10—O2	124.84 (19)	O6—C22—O5	125.0 (2)
O3—C10—C9	123.04 (18)	O6—C22—C21	122.62 (18)
O2—C10—C9	112.13 (17)	O5—C22—C21	112.33 (16)
O2—C11—C12	107.24 (16)	O5—C23—C24	107.44 (17)
O2—C11—H11A	110.3	O5—C23—H23A	110.2
C12—C11—H11A	110.3	C24—C23—H23A	110.2
O2—C11—H11B	110.3	O5—C23—H23B	110.2
C12—C11—H11B	110.3	C24—C23—H23B	110.2
H11A—C11—H11B	108.5	H23A—C23—H23B	108.5
C11—C12—H12A	109.5	C23—C24—H24A	109.5
C11—C12—H12B	109.5	C23—C24—H24B	109.5
H12A—C12—H12B	109.5	H24A—C24—H24B	109.5
C11—C12—H12C	109.5	C23—C24—H24C	109.5
H12A—C12—H12C	109.5	H24A—C24—H24C	109.5
H12B—C12—H12C	109.5	H24B—C24—H24C	109.5

### Hydrogen-bond geometry (Å, º)

**Table:**

*D*—H···*A*	*D*—H	H···*A*	*D*···*A*	*D*—H···*A*
N3—H3*C*···N2^i^	0.86	2.44	3.243 (3)	157
N3—H3*B*···O6^ii^	0.86	2.62	3.396 (2)	150
N1—H1*B*···N4	0.86	2.44	3.255 (3)	159
N1—H1*A*···O3^iii^	0.86	2.63	3.394 (3)	149

Symmetry codes: (i) *x*−1, *y*, *z*+1; (ii) *x*, *y*+1, *z*; (iii) *x*, *y*−1, *z*.
